# Signaling Pathways of Receptors Involved in Platelet Activation and Shedding of These Receptors in Stored Platelets

**DOI:** 10.15171/apb.2019.005

**Published:** 2019-02-21

**Authors:** Asra Amelirad, Karim Shamsasenjan, Parvin Akbarzadehlaleh, Davoud Pashoutan Sarvar

**Affiliations:** ^1^Immunology Research Center, Tabriz University of Medical Sciences, Tabriz, Iran.; ^2^Department of Pharmaceutical Biotechnology, Faculty of Pharmacy, Tabriz University of Medical Sciences, Tabriz, Iran.; ^3^Asadabad School of Medical Sciences, Asadabad, Iran.

**Keywords:** ADAM Proteins, Metalloproteases, Platelet activation, Platelet membrane glycoproteinsins, Platelet storage pool deficiencycy, Platelet transfusion

## Abstract

All cells encounter various signals coming from the surrounding environment and they need to receive and respond to these signals in order to perform their functions. Cell surface receptors are responsible for signal transduction .Platelets are blood cells which perform several functions using diverse receptors. Platelet concentrate is one of the most consumed blood products. However, due to the short lifespan of the platelets and platelets damage during storage, we face shortage of platelet products. One of the damages that platelets undergo during storage is the loss of surface receptors. Since cell surface receptors are responsible for all cell functions, the loss of platelet receptors reduces the quality of platelet products. In this study, we reviewed the important receptors involved in platelet activation and their associated signaling pathways. We also looked at the platelet receptors that shed during storage and the causes of this incident. We found that GPIbα, P-selectin, CD40 and GPVI are platelet receptors that fall during platelet storage at room temperature. Considering that GPVI and GPIbα are the most important receptors which involved in platelet activation, their shedding can cause decrease in platelet activation after transfusion and decrease thrombus consistence. Shear stress and platelet contact with the container wall are among the mechanisms discussed in this process, but studies in this area have to be continued.

## Introduction

Platelets are the smallest blood cells (~2.5 μm) and human adults approximately have 1 trillion platelets in circulation that are turned over every 8–10 days.^1,2^ They are metabolic active cells, and are seen in numerous functional organelles, highly organized cytoskeleton, vast array of receptors, and many secretory granules.^3^ Platelets are formed from mature megakaryocytes and arisen from the long tube-like developed cytoplasmic extension called proplatelets in particular platelets develop process. After release of platelets, the megakaryocyte nucleus, its envelope, and its neighbor cytoplasm, usually remains in the marrow, and finally, phagocytized by macrophages.^4^ Megakaryocytes are derived from hematopoietic stem cells (HSCs) in bone marrow.^5,6^ Bone marrow microenvironment contains cellular and acellular compartments. Cellular compartment contains HSCs, mesenchymal stem cells (MSCs), and some other kinds of stromal cells. On the other hand, acellular compartment includes scaffold proteins known as extra cellular matrix.^7^ HSCs are able to produce various blood cells.^5^ MSCs which recognized as main components of stromal cell niches support HSCs homing, proliferation, self-renewal, and differentiation in the bone marrow. Cheng et al^8^ demonstrated under co-culture conditions, MSCs are able to support megakaryocyte differentiation and platelet formation from CD34b HSC.^9^ Although platelets are well-known because of their essential role in homeostasis and thrombus formation, they have many different functions. Platelets release pro-inflammatory and anti‐inflammatory, angiogenic factors, and microparticles into the circulation and play serious roles in the host defense, inflammation, angiogenesis, tumor growth and metastasis.^10-14^ Platelet receptors are responsible for all these functions and their density and affinity controls the cell function directly. Platelets are unable to perform these functions in the absence of their receptors. Disorder of platelet receptors were first described by Glanzmann^15^ in 1918and Bernard & Soulier^16^ in 1948. After the recognition of these disorders, structure and functions of platelet receptors were extensively studied. In the loss of ligand binding and in shear stress status, cell surface receptors are down regulated. One of the receptors down regulation mechanisms is ectodomain shedding. In this mechanism, protein will break in a near location to external surface of membrane layer.^17-19^ One of the conditions in which platelets experience shear stresses in storage conditions. In such situation, some of the platelet surface receptors are involved in surface deduction by shedding.^20,21^ Receptor shedding in the stored platelet reduce platelet quality and is one of the platelet storage lesions.^22^ In the following, we introduce:

- Platelet receptor signaling with a focus on signaling pathways associated with platelet activation
- Integrin activation (one of the key events in platelet activation)
- Basic mechanism of ectodomain shedding in platelets
- Receptor shedding during platelets storage

## Platelet receptor signaling

Platelets perform their functions inside the vessels by using 3 types of signals: inhibitory, activation and negative feedback.^23^

### Inhibitory signals

Inhibitory signals allow platelets to circulate in a resting state. Platelets are activated even in the absence of activation signals. In healthy vessels, endothelium expresses fundamental forms of nitric oxide synthase (NOSIII) and cyclo-oxygenase-1 (COX-1), which produce the vasoactive hormones NO and prostacyclin (PGI2), respectively. Both NO and PGI2 are co-released by endothelial cells and act in synergy to inhibit platelet activation, thereby limiting thrombosis. Nitric oxide activates soluble guanylyl cyclase, present in the cytosol, causing an increase in intracellular cGMP from GTP. An immediate consequence of increasing cGMP is direct activation of protein kinase g (PKg). The activated PKg reduces the intracellular calcium and cell activation by phosphorylation of several targets. PGI2 is able to bind with the prostacyclin receptor (IP) on the surface of platelets. Activation of the IP receptor on the surface of platelets induces production of cAMP, causing activation of PKa, the subsequent inhibition of several pathways including PKc activation, calcium release, and platelet inhibition. In addition to the NO and PGI2, CD39 is another mechanism of inhibitory function in the vascular endothelium. In platelet membrane, CD39 hydrolyze endothelium and red cells secreted ADP to AMP and adenosine. Adenosine activates the Gs-coupled adenosine receptor, and leads to inhibition of platelet through elevation of cAMP.^23-28^

### Activatory signals

Among the various platelet receptors that are known, 2 groups of platelets receptors are involved in platelets activation: adhesion receptors and G protein-coupled receptors. Glycoprotein (GP) Ib-IХ-V, GP Ia/IIa and GPVI are 3 important adhesion receptors, which play a role in platelet activation.^29^ In the following, we explain the pathways for platelet activation (Table 1).

**Table 1:** Platelet receptors

**Receptor family**	**Example**
Integrins	α2β1^30^, α5β1^31^, αIIbβ3^32^
Lucine rich repeat family	GPIb-IX-V^33^
Selectin s	CD62P^34^, CELEC2^35^
Tetraspanins	CD63^36^
Transmembrane receptors	P2Y_1_ and P2Y_12_^37^
Prostaglandin receptors	Prostacyclin receptors, thromboxane receptors^38^
Lipid receptors	PAF receptors^39^
Immunoglobulin superfamily receptors	GPVI, CD32^40^
Tyrosine kinase receptors	Thrombopoietin receptors^41^
Miscellaneous platelet membrane receptors	Serotonin receptors^42^

### VWF/GPIb-IX mediated platelet activation

Von will brand factor is a multi-subunit glycoprotein that circulates in plasma. It is synthesized by endothelial cells and megakaryocytes, that released through a regulated pathway after storage in endothelial Weibel-Palade bodies and platelet α granules.^43,44^ The mature von Willebrand factor (VWF) subunit has 2,050 residues with multiple A-, B-, C-, and D-type domains. The A1 domain contains binding site for platelet GPIbα.^45,46^ GPIb-IX-V is composed by 4 distinct trans membrane proteins: 2 chains of GPIbα (135 kDa), 2 GPIbβ (26 kDa), 2 GPIX (20 kDa) and 1 GPV (82 kDa). These proteins are encoded by 4 different genes and belong to lucine rich family that map to chromosomes 17q12 (*GPIbα*), 22q11.2 (*GPIbβ*), 3q29 (*GP5*) and 3q21 (*GP9*), respectively. GPIb-IX-V expresses on the platelet membrane exclusively. There are approximately, 25 000 copies of this receptor per platelet. When blood vessels are disrupted, circulating platelets adhere to exposed subendothelial surfaces through interactions of platelet GPIbα with VWF, which is immobilized on collagen fibers. This is the first step in a cascade of adhesion and signaling events that produce a hemostatic plug at the injured site. Platelets tether to and roll on immobilized VWF, but do not adhere firmly.^47-50^ The interaction between VWF and GPIb-IX-V not only mediates transient platelet adhesion but also initiates a signaling cascade result in platelet integrin α_IIb_β_3_ activation and outcome stable platelet adhesion, spreading, and aggregation.^51,52^ Platelet activation via VWF/GPIb-IX occurs only in high flow rates. Several intracellular signaling molecules and pathways in GPIb-IX-mediated platelet activation have been included: the phosphatidyl inositol 3-kinase (PI3-kinase) protein kinase b (AKT) pathway, the mitogen-activated protein kinase (MAPK) pathways, and the FCRγ-SYK/PLCγ2 pathway. Nevertheless, the detailed mechanism of this process remains unclear. There have been conflicted reports regarding to the role of spleen tyrosine kinase (SYK) in GPIb signaling.^53,54^

### Collagen/GPVI mediated platelet activation

Collagens are the most numerous proteins in the subendothelial extracellular matrix and in addition are essential in platelet adherence and platelet plug establishment to providing mechanical strength to the blood vessel walls.^46^ Among various collagen types only fibrillar collagen type I, III, V, VI and nonfibrillar collagen type IV and VIII are thrombogenic. Although platelets have various receptors for collagen, the integrin α2β1 and GPVI are the most important collagen receptors on platelet surface for binding to collagen and activation of platelets.^29^ GPVI (62 kDa) is a type of I transmembrane receptor expressed on platelets and megakaryocytes exclusively. It comprises 2 Ig extracellular domains formed by disulphide bonds, a mucin-like stalk and a short 51-AA cytoplasmic tail.^55^ The GPVI cytosolic tail contains recognized sequence motifs for binding to calmodulin and the SH3 domain of Src family tyrosine kinases positively charged arginine in transmembrane region of GPVI that formed a disulfide‐linked with FC receptor (FCR) γ‐chain. Each FCR γ‐chain contains one copy of an immunoreceptor, tyrosine‐based activation motif (ITAM) that undergoes phosphorylation on 2 conserved tyrosines upon crosslinking of GPVI, leading to binding and activation of the tyrosine kinase SYK, which phosphorylates downstream targets, such as LAT and SLP-76. This subject induces the establishment of a signaling complex, including LAT, SLP-76, Bruton tyrosine kinase (BTK), Grb2-related adaptor downstream of Shc (GADs), and phospholipase Cγ (PLCγ) 2, which further activates PLCγ2. PLCγ2 activation leads to catalyzes the formation of inositol 1, 4, 5-trisphosphate (IP3) and diacylglycerol (DAG) from phosphatidylinositol 4, 5-bisphosphate. DAG and IP3 formations activate protein kinase C (PKC) and Ca^2+^ release from intracellular stores, respectively. This result in the integrin-mediated adhesion of platelets and some cellular activation, which leads to the deliver and production of some platelet agonists such as ADP, TxA2, and thrombin acting via GPCRs.^48,55-57^

### CLEC-2/podoplanin mediate platelet activation

CLEC-2 is a C-type lectin-like type ii transmembrane receptor.^58^ in platelet binding of rhodocytin (exogenous ligand) and podoplanin (endogenous ligand) to CLEC-2 in platelets surface triggers a novel platelet-signaling pathway.^59^ Similar to collagen receptor glycoprotein (GP) VI/FCRγ-chain complex, CLEC2 ligand binding leads to tyrosine phosphorylation in the cytoplasmic tail of CLEC-2, which promotes the binding of SYK, subsequent activation of PLCγ2, and platelet activation and aggregation.^60,61^ Podoplanin is expressed on the surface of defined types of cancer cells and various normal cells such as kidney podocytes, type I lung alveolar cells, fibroblastic reticular cells in lymph nodes, and lymphatic endothelial cells. CLEC‐2‐deficient platelets displayed normal adhesion under flow conditions, but further thrombus formation was severely impaired in vitro and in vivo.^62^ Considering that, Podoplanin is expressed on the surface of tumor cells. Therefore, platelet activation by CLEC-2/podoplanin interaction facilitates tumor metastasis.^59,63^

### Platelet activation and signaling mediated by G protein-coupled receptors

Platelet signaling begins with activation of platelet receptors by agonists such as PAF, collagen, thrombin, ADP, TxA2 and epinephrine. Except collagen, which is described and acts as the first line of hemostasis in platelets, other agonists work through one or more members of G-coupled receptor superfamily.^64^ Through the activation of G protein–mediated signaling pathways, they can further increase their own formation and release; thus they acting as positive-feedback mediators that amplify the initial signals to ensure the rapid activation and recruitment of platelets into a growing plaque.^57^ G protein–coupled receptors (GPCRs) compose one of the largest families of membrane proteins involved in intracellular signaling. All GPCRs share a common serpentine structure of seven transmembrane‐spanning domains, with an extracellular n‐terminus and an intracellular c‐terminus. GPCRs are so‐named because they are physically associated with heterotrimeric G proteins. Each G protein is composed of α, β, and γ subunit. After receptor ligation α subunit dissociates from the βγ subunits, which allows exposure of surfaces on both α and βγ subunits for interaction with effector proteins. G proteins are generally classified into 4 families: G_S_, G_i_/Go/Gz, Gq/G_11_, and G_12/13_. Each of them is coupled to selective receptors and downstream effectors. Platelet activation via G protein-coupled receptors involves 3 major G protein-mediated signaling pathways that are initiated by the activation of the G proteins, Gq, G_13_, and G_i_. Although, in the absence of Gq-, G_13-_, or G_i_ -mediated signaling, some platelet activation can occur, efficient activation of platelets in vitro and in vivo requires all 3 G protein–mediated signaling pathways. TP (thromboxane a2 receptor), PAR3, PAR4 and PAR1 (thrombin receptors) which are coupled to Gq, and G_13_, P2Y1 (ADP receptor) are coupled to Gq, and P2Y12 (ADP receptor) are coupled to G_i_. Gq transmit cellular signals commonly through its interaction and activation of PLCβ. Gq signaling is necessary for GPCR-stimulated platelet granule secretion, integrin activation, and consequent platelet aggregation. Gq signaling is necessary but insufficient for platelet aggregation and induced by ADP and optimal platelet response induced by TXA_2_ or low dose thrombin. Gq also needs Gi-coupled to carry out these activities.^48,57,65-69^ Gα13 knockout platelets show reduction in granule secretion and unstable platelet aggregation induced by TxA2 analog U46619. In addition, platelet aggregation induced by low dose thrombin in Gα13 knockout platelets is decrease.^70^ All GPCR activity in the platelets depends on the G protein, as the knock‐out of individual G proteins has been sufficient to disrupt platelet responses to receptor agonists (Table 2).^65^

**Table 2:** G protein-coupled receptors on human platelets

**G protein**	**Agonist**	**Receptor**	**Effector/signaling**	**References**
Gq	Thrombin	PAR1	Phospholipase C/Ca^++^ release, PKC activation	^71,72^
Gq	Thrombin	PAR4	Phospholipase C/Ca^++^ release, PKC activation	^73-75^
Gq	ADP	P2Y1	Phospholipase C Ca^++^ release, PKC activation	^76,77^
Gq	TxA2	TP	Phospholipase C, Ca^++^ release, PKC activation	^69^
G13	Thrombin	PAR1	Rho activation, actin remodeling	^78^
G13	TxA2	TP	Rho activation, actin remodeling	^78,79^
Gi2	ADP	P2Y12	↓ cAMP, PI3Kγ activation	^80,81^
G_s_	PGI_2_	IP	↑ cAMP	^82^
G_z_	Epinephrine	α_2A_ adrenergic	↓ cAMP	^75,83^

PAR1, Protease activated receptor; TP, Thromboxane receptor; IP, Prostacyclin receptor; PI3, Phosphoinositide 3-kinase

### Negative feedback signals

Maintenance of the proper balance between platelet activation and platelet inhibition is critical because disruption of this balance can cause thrombotic or bleeding disorders, respectively.^84^ Following initiation of platelet activation and thrombus formation, to control excess clot production, a number of negative regulator signals prevent the activation of more platelets. A number of endogenous inhibitory mechanisms are inhibitory receptors on the surface of platelets e.g., platelet endothelial cell adhesion molecule-1, Intracellular inhibitory receptors e.g., Liver X Receptor α and β and Emerging inhibitory pathways e.g., semaphorin 3A and junctional adhesion molecule-a.^85-89^

## Key events in platelet activation

Platelet activation through agonists-GPCRs Signaling pathways induces platelet-shape change, degranulation, and integrin α_IIb_β_3_-mediated aggregation.^57,90^ In this section, we describe the Integrin activation.

### Integrin activation

Integrins are a widely family of heterodimeric transmembrane receptors that are connecting extracellular ligands to mediate cell adhesion α_2_β_1_ and intracellular signaling pathways.^91^ Integrins composed by α- and β-subunits, which are non-covalently linked to each other. Both subunits traverse the plasma membrane and terminate short cytoplasmic domains.^92^ α_IIb_β_3_ (fibrinogen receptor), α_V_β_3_ (vitronectin receptor), (collagen receptor), α_5_β_1_ (fibronectin receptor), and α_6_β_1_ (laminin receptor) are expressed in platelet. Among these integrins, α_IIb_β_3_ is the most abundant integrin in the platelets. α_IIb_β_3_ is normally kept in a low affinity state in circulating platelets, but transforms into a high affinity state following platelet activation.^93^ This transformation allows α_IIb_β_3_ to bind Arg-Gly-Asp (RGD) sequence in their ligands. Activation of α_IIb_β_3_ is tightly regulated through a process termed ‘inside‐out signaling’. It has been shown that inside-out signaling requires the binding of talin and kindlins to the cytoplasmic domain of β_3_. In addition, recent studies suggest that CalDAG-GEF1 and its downstream target, Rap1, plays an important role in inside-out signaling.^48,91^ Conversely, the interaction between integrins and their various ligands (fibrinogen, VWF, vitronectin and fibronectin) induces outside-in signals across the membrane that allows αIIb β_3_ clustering, e.g. during platelet aggregation. One of the earliest events occurring during integrin outside-in signaling is the tyrosine phosphorylation of specific substrates. The Src family of kinases (SFKs) has a dominant effect in these phosphorylation events. Src was originally proposed to be constitutively associated with the β3 integrin C-terminal tail in an inactive conformation of resting platelets via its SH3 domain. Upon ligand binding to αIIbβ3 and integrin clustering, protein phosphatases relieve the inhibitory Src phosphorylation with dissociation of C terminal Src kinase from β3, permitting Src activation. Src activation results in activation of the tyrosine kinase Syk. Syk substrates contain important outside-in effectors, including the RhoGEFs Vav1 and Vav3, and the SH2- containing leukocyte protein of 76 kDa (SLP-76). In addition, SFKs phosphorylate a host of signaling and cytoskeletal-associated proteins in platelets, including phospholipase Cγ2 (PLCγ2), focal adhesion kinase (FAK), and degranulation promoting adapter protein (ADAP), resulting recruitment and/or activation of these proteins.^94^ Activated FAK modulates the activity of a broad range of downstream signaling proteins, including PI3- Kinases, PLC–γ as well as a number of small GTPases such as Ras, Rac, and Rho.^95^ ADAP interactions with talin and kindlin promote platelet integrin αIIbβ3 activation and stable fibrinogen binding.^96^ In general, inside-out signaling activates the ligand binding function of integrins and outside-in signaling mediates cellular responses induced by ligand binding to integrins leading to cell spreading, granule secretion, retraction, migration, and proliferation.^97^

## Platelet receptor shedding

### Role of shedding in platelet function

Cells membrane receptors are common beginners in cell signaling. Therefore, receptors density and affinity controls the cell function.^18^ However, there are extensive information in literature about activation of platelet receptor.^19^ Nevertheless, downregulation of these receptors have not been recognized properly. One of the external cells receptors downregulation mechanisms is ectodomain shedding. In this mechanism, protein breaks near the external surface of membrane layer and the isolated ectodomain will release into the plasma.^19,98,99^ Disjunction part can be operational or use as a private biomarker of platelet.^100^ Ectodomain shedding can be a useful mechanism in abnucleus cells such as platelets because in these cells control of receptor surface through regulation of gene expression has an inconspicuous role.^18,100^ Ectodomain shedding can have other roles in addition to controlling levels of superficial proteins. For instance, the capacity of platelets to form filopodia and lamellipodia and spread on a VWF and/or collagen matrix requires the dynamic breaking of existing receptor/matrix ligand bonds and formation of new receptor matrix/ligand interactions at the tips of filopodia or the spreading lamellipodial edge. One mechanism for how this could occur is through receptor ectodomain shedding.^19^ According to the several investigations, 69 platelet membrane proteins have been identified in activated protein supernatant which are prone for shedding. It has been observed that shedding occurs in 12 membrane proteins out of these 69 proteins including semaphorin7a, CD84, GPV, amyloid beta A4, GPIbα, TLT-1, P-selectin, JAMA-1, CD40-L, semaphorin 4D, PECAM and GPVI. There are limited surveys on the remaining 57 membrane proteins.^101-111^

### Sheddase activation

Two members of a disintegrin and metalloprotease family (ADAMs) called ADAM10 and ADAM17 accomplish ectodomain shedding mechanism.^19,112^ ADAM10 and ADAM17 consist of a pro-peptide domain, catalytic domain, disintegrin domain, regulatory Cys-rich domain, transmembrane region, and cytoplasmic tail. They may be regulated by (*a*) cysteine-switch, where a free sulfhydryl in the pro-peptide domain interacts with the active-site metal ion inhibiting the enzyme, or (*b*) intracellular signals, required for ADAM mediated ectodomain shedding. ADAMs have a significant role in superficial proteins ectodomain shedding including growth factor, receptors and their ligands, cytokines and adhesion molecules.^19^ ADAMs will be active after cell activation or connecting to receptors which are able to shedding potentially. However, ADAMs basic activation mechanism is complex and has not been recognized completely. In some studies on the activation mechanism of these sheddase, it has been observed, for example, that (*a*) thiol modifying agents are able to activate ADAMs directly, (*b*) high amounts of ASA cause GPIbα shedding by ADAM17, or (*c*) calmodulin restrain which attached to cytoplasmic sequences of some membrane receptors cause the shedding of these receptors. Actually, internal cell signals as external ones are able to cause induction shedding.^18^

Various studies in recent years have shown that calmodulin is connected to internal cell domain of GPVI, GPV and GPVIβ in circulate platelets and activation of platelets by different agonist causes calmodulin separates from internal cell domain of these proteins. Also, it has been observed calmodulin inhibition causes external domain shedding of GPV and GPVI. However, GPIbβ is not affected by shedding in calmodulin inhibition. On the other hand, GPIbα which is connected to GPIbβ by disulfide and non-covalent bond is affected by shedding. It seems that connected calmodulin to GPIbß has a restrain effect on GPIbα shedding and this is only report that shows a receptor has experienced shedding restrain by another cytoplasmic sequences receptor.^19,98^

### Shedding in stored platelet Receptor

Platelet concentrates (PCs) are the most vulnerable blood products with the shortest shelf life.^113,114^ In recent years, requirement to platelet products is being increased due to increment of the patients who are being treated in bone marrow suppression. At the moment platelet are not only being used to control or prevent bleeding but also being increasingly used as a source of growth factors in tissue repair, wound redressing, and skin rejuvenation.^115^ Using autologous platelet rich products including platelet lysate, platelet rich plasma and platelet rich fibrin for MScs expansion become more general employing autologous platelet rich products for MScs expansion is a convenient, non-toxic, safe and cheap therapeutic method that promotes using MScs for cell therapy. These 3 products contain a variety of growth factors including platelet-derived growth factor, fibroblast growth factor, insulin-like growth factor, transforming growth factor, platelet factor 4, and platelet-derived epidermal growth factor. These growth factors enhance and accelerate MSc.^116^ however, the short half-life of platelets has caused these products have the most wasting amounts among blood products; for example, in Canada platelet’s half-life is about 5 days and 30% of them will be out of reach.^117^ PSL (platelet storage lesion) is one of the main reasons of platelets short half-life. PSL explains structural and functional changes in platelets from bloodletting until the platelet transfusion.^115^ Shedding of platelets surface receptor during storage is one of the PSL. The receptor shedding has an obvious difference with other processes such as the losing of receptor surface through internationalization, release of micro particles, and secretion process. In the secretion process, proteins from platelet storage granules releases.^110,118^

PSL accelerates clearance of platelet after transfusion and has connections with various elements including media, agitation method, bag materials, storage temperature, and so on. Unlike the erythrocyte, platelets are kept between 20-24^°^C.^117,119-123^ However, keeping the platelets in this range of temperature increases the percentage of bacterial infection and decreases hemostatic activity. Refrigerated platelets do not have these problems, however they eliminate from blood circulation shortly after injection. Desialyation of platelets is considered as one of the mechanisms for rapid elimination of refrigerated platelets that purge them by liver macrophage or hepatocyte through recognition of exposed glycan. Notably, desialylated GPIbα also shows increased susceptibility toward ADAM17-mediated metalloproteolysis.^118,124^

Beside the platelet activation, there are some other materials such as NEM (N-ethylmaleimide), W7 (N-(6-Aminohexyl)-5-chloro-1-naphthalenesulfonamide), PMA (PKc activator phorbol-12-myristate-13-acetate), ASA (acetylsalicylic acid), and CCCP (mitochondrial-targeting compound carbonyl cyanide 3-chlorophenylhydrazone) which can induce GPIbα shedding. This shedding cause production of a part called glycocalcin, a soluble n-terminal 130 kDa ectodomain fragment, which is further processed in the plasma by proteases. Glycocalcin is a private biomarker at PSL and refrigerated platelets.

Since shedding is a proteolytic reaction that is dependent on enzyme, controlling of enzyme or substrate is inevitable for restraining shedding. In the case of controlling enzymes for GPIbα shedding, injection of GM6001 or MAPK p38 (which control ADAM17 enzyme) in the storage platelets decreases shedding of this glycoprotein and improves the result of platelet transfusion. Moreover, monoclonal antibody 5G6, which acts as a substrate controller, connects to GPIbα cleavage and prevents enzymes to connect to GPIbα. Thus, there is a close connection between ectodomain shedding and clearance of injected platelets. The reason can be described as follows: decrease of GPIbα surface affected by shedding in storage platelets is effective on adhesion strength of injected platelets under the increase of venous rate share. These platelets have lower functional power and will be eliminated from blood circulation rapidly.^125,126^ GPV is another glycoprotein that experiences shedding during refrigerating of platelets. It seems that GPV shedding is controlled by both ADAM17 and ADAM10, and it is a dependent mechanism on thrombin. On the other hand, GPIbα shedding only depends on ADAM17. The other platelet surface glycoproteins including GPVI, GPIbβ, GPIX and glycoprotein IIb*/*IIIa are not affected by refrigerated storage.^127^

GPIbα shedding is also occurred in storage platelets in room temperature. In room temperature, P-selectin, CD40 and GPVI are also involved in surface deduction by shedding mechanism beside GPIbα. The amount of expression and shedding GPIbα and GPVI have a close correlation, and at the same time, there is a negative connection between these 2 glycoproteins with P-selectin and CD40 expression measure. However, all of their sheddings are increased during storage.^126^ Similar to GPIbα, signaling depended on GPVI has a significant role in cross-linking of receptor. This receptor shedding decreases cell signaling time and causes decreasing in platelet activation and secretion. In addition, these glycoproteins’ shedding decrease thrombus consistence and make an easy establishment of thromboembolus.^19^

GPVI shedding is more seriously regulated than GPIbα, and different shedding mechanisms may be involved GPVI shedding is induced by GPVI ligands such as collagen, convulxin and CRP. However, under conditions where GPVI is completely lost, GPIbα is detected on platelets. This difference in regulation reflects the fact that GPVI directly binds to calmodulin at the cytoplasmic domain whereas the cytoplasmic domain of GPIbα does not bind to calmodulin.^128^ Levels of GPVI shedding are higher in stored platelets in compared with non-stored samples activated under the effect of agonists such as Calcium Ionophore. Either interplaying of platelet surface with the container walls during storage or induced shear stress under long-period agitation might play role in the excessive shedding of GPVI during platelet storage.^126^ Shedding of 2 key platelet receptors, glycoprotein (GP) Ibα and GPVI, after exposed to the non-physiological high shear stress environment exists in blood contacting medical devices and stenotic blood vessels has also been reported.^129^

## Conclusion

All cells are constantly exposed to a variety of extracellular signals. The cells surface receptors are responsible for responding to these signals and, in this way, they control all cell functions. In this paper, we first describe those receptors and signaling pathways, which lead to the activation of platelets, and then explain the ectodomain shedding, which is one of the methods for controlling platelet surface receptors. Two main groups of receptors play roles in the activation of platelets: adhesion receptors and G protein-coupled receptors. However, the initial signaling mechanisms of these 2 receptor groups are different. They ultimately converge into common intracellular signaling events. In particular, almost all agonists induce activation of PLC. The cells use different mechanisms to control the level of their receptors. One of these mechanisms is ectodomain shedding which can be a useful mechanism in no-nucleus cells such as platelets because in these cells control of receptor surface through regulation of gene expression has an inconspicuous role. Regularly, shedding has been occurred after platelets activation, but it has been reported that it has also happened in circulating platelets. However, the platelet receptor shedding does not occur only in vivo. Several studies have suggested the presence of platelet receptors shedding in shear stress conditions, such as storage condition. GPIbα, P-selectin, CD40 and GPVI are induced by ectodomain shedding mechanism in storage bag. As we know, GPVI and GPIbα are the most important receptors, which involved in platelet activation. Therefore, their shedding can reduce cell signaling time and finally, cause decrease platelet activation and secretion. Moreover, these glycoproteins’ shedding decreases thrombus consistence and makes an easy establishment of thromboembolus. In addition to shear stress, the platelet contact with the container wall, as another possible cause of this mechanism, has been raised, but studies in this area have to be continued. Thus, finding the main reasons for platelet shedding, which is one of the PSL cases in platelet storage conditions, helps to find a solution to prevent this mechanism or reduce its rate and speed, and ultimately to improve the quality of the storage platelets. Based on the studies reviewed here, there are 2 main possibilities for improving shedding. First method is change the agitation method and its revolution speed and second is using different platelet storage bag. Thus, investigation the effects of these 2 mode on shedding decrease is an interesting subject for further studies.

## Ethical Issues

Not applicable.

## Conflict of Interest

The authors declare that they have no conflict of interest.

## Acknowledgments

The authors would like to thank Mr. Omid Amelirad (Department of Mechanical Engineering, Sharif University of Technology, Tehran, Iran) and Ms. Sarah Aqmasheh (Department of Immunology, Tabriz University of Medical Science, Tabriz, Iran) for helping us in editing the draft article.
